# Separation of microalgae from bacterial contaminants using spiral microchannel in the presence of a chemoattractant

**DOI:** 10.1186/s40643-024-00746-8

**Published:** 2024-04-13

**Authors:** Leticia F. Ngum, Y. Matsushita, Samir F. El-Mashtoly, Ahmed M. R. Fath El-Bab, Ahmed L Abdel-Mawgood

**Affiliations:** 1 Institute of Basic and Applied Sciences, Biotechnology Program, Egypt-Japan University of Science and Technology, Alexandria, 21934 Egypt; 2 Institute of Basic and Applied Sciences, Nanoscience Program, Egypt-Japan University of Science and Technology, Alexandria, 21934 Egypt; 3 Mechatronics and Robotics Engineering Department, Egypt-Japan University of Science and Technology, Alexandria, 21934 Egypt

**Keywords:** W-shaped cross-section, CO_2_ laser ablation, Glycine, Separation efficiency, Removal ratio

## Abstract

Cell separation using microfluidics has become an effective method to isolate biological contaminants from bodily fluids and cell cultures, such as isolating bacteria contaminants from microalgae cultures and isolating bacteria contaminants from white blood cells. In this study, bacterial cells were used as a model contaminant in microalgae culture in a passive microfluidics device, which relies on hydrodynamic forces to demonstrate the separation of microalgae from bacteria contaminants in U and W-shaped cross-section spiral microchannel fabricated by defocusing CO_2_ laser ablation. At a flow rate of 0.7 ml/min in the presence of glycine as bacteria chemoattractant, the spiral microfluidics devices with U and W-shaped cross-sections were able to isolate microalgae (*Desmodesmus* sp.) from bacteria (*E. coli*) with a high separation efficiency of 92% and 96% respectively. At the same flow rate, in the absence of glycine, the separation efficiency of microalgae for U- and W-shaped cross-sections was 91% and 96%, respectively. It was found that the spiral microchannel device with a W-shaped cross-section with a barrier in the center of the channel showed significantly higher separation efficiency. Spiral microchannel chips with U- or W-shaped cross-sections were easy to fabricate and exhibited high throughput. With these advantages, these devices could be widely applicable to other cell separation applications, such as separating circulating tumor cells from blood.

**Graphical Abstract:**

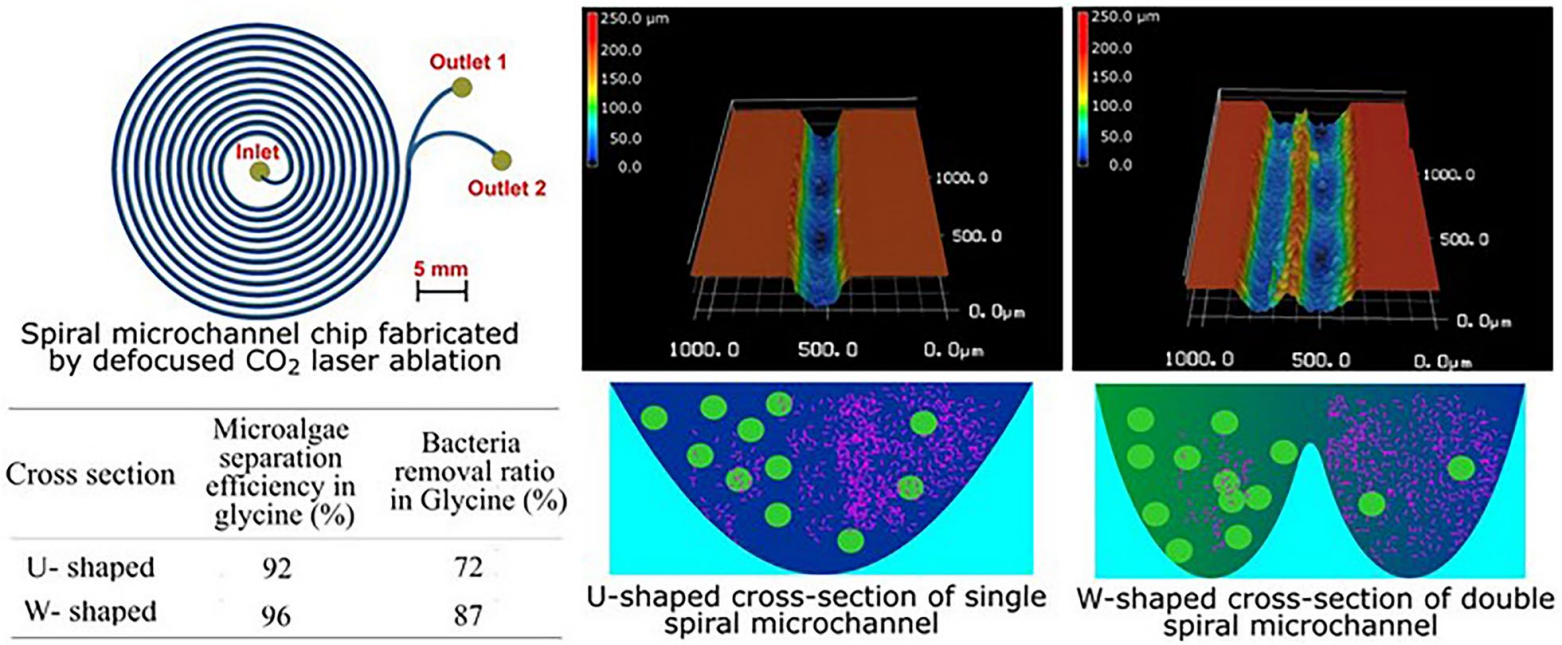

## Introduction

Microalgae are photosynthetic, unicellular microorganisms that thrive in freshwater and marine environments and often comprise the base of aquatic food chain in seas. In recent years, microalgae has attracted significant attention worldwide as a potentially valuable resource for biomass cultivation (Ruiz et al. 2016). This is due to microalgae's ability to absorb CO_2_ through photosynthesis (Borowitzka 2013), their greater biomass productivity compared to terrestrial plants (Wan et al. 2015), and their use in feedstocks to produce high-value products like food, cosmetics and renewable energy, thereby meeting global demand (Yuan et al. 2017). The aforementioned industrial potential, typically achieved by growing a single algal strain with a high proportion of target product, is only possessed by a few unique species (Kim et al. 2021). Therefore, it is crucial to separate microalgae strains with desired characteristics from their natural habitats to facilitate productivity in laboratory research and effective commercial applications.

Microalgae cultures are commonly contaminated by invasive bacteria, fungi and other microalgae species during cultivation and collection (Patil et al. 2008). Hence, purification processes to remove microalgae from undesirable microorganisms before downstream storage is required to ensure high-efficiency production of biomass. This biomass can be a potential source for downstream manufacturing such as bioplastics and bioactive film production. (Morales-Jiménez et al. 2020) used extracellular biopolymers from Nostoc sp. and Porphyridium purpureum biomass to produce transparent and flexible bioactive films with antifungal properties that can be utilized for food and cosmetic packaging. Other application is the biofuel production due to its high lipid and carbohydrate content (Morales-Jiménez et al. 2021). Other bio-based products such as asthanxanthin produced from *Haematococcus pluvialis*, phycocyanobilin from *Spirulina* sp., and ß-1,3-Glucan from Chlorella sp., which are used to produce cosmetic products with high antioxidant and anti-inflammatory contents (Mourelle et al. 2016).

Standard methods for reducing, isolating and purifying microalgae cultures from bacterial contaminants include streaking on a selective medium using agar plate culture, serial dilution (Syed et al. 2018), micro pipetting (Kim et al. 2021), centrifugation and filtration (Yuan et al. 2019). However, the streaking methods are ineffective for microalgae because algal cells form identical green colonies are difficult to differentiate (Kim et al. 2021). Micro-pipetting techniques are highly labor-intensive and can lead to cell damage (Spilling 2017). In addition, the centrifugation method is time-consuming and leads to the destruction of the analytes of interest due to mechanical stress caused by high-speed centrifuge rotation and the filtration methods can lead to filter clogging (Yu et al. 2015). Hence, the shortcomings associated with these conventional technologies have led to the development of more sensitive, less time-consuming technologies such as microfluidics, which can be used to improve the separation of microalgae cultured cells from bacterial contaminants, thereby eliminating contamination from microalgae cultures.

Cell sorting, cell detection, cell preparation, and other essential activities can be performed on a single-chip system using microfluidics technology. Microfluidics is a technique that handles small amounts of fluids utilizing tiny channels with sizes ranging from tens to hundreds of micrometres. Microfluidics has evolved as an exciting and essential tool in biology, medicine, chemistry, agriculture, food, environment, navigation, and many other fields (Bodénès et al. 2019) because of its low fabrication cost, ease of operation, high throughput, lack of cell damage, and the possibility of coupling with microscopy (Kang et al. 2018). Despite the advantages associated with microfluidics, the clogging of microchannels is a significant problem during cell separation. This clogging may be due to the aggregation of cells on the channel wall as a result of a high concentration of cells in the microchannels, which prevents the movement of cells in the microchannel (Kang et al. 2018).

Based on the operation concept, microfluidic techniques can be into two categories, active and passive. In active separation methods such as dielectrophoresis (DEP) (Bakhshi et al. 2022), magnetophoresis (Hejazian et al. 2015), and acoustophoresis (Olm et al. 2019), cells are separated by external forces.

Conversely, passive approaches do not require external forces to separate cells. Passive technologies are more prevalent than active ones due to their inexpensive cost of manufacture, high throughput, and straightforward channel designs. These passive techniques include inertial microfluidics (Chung 2019), deterministic lateral displacements (DLD) (McGrath et al. 2014), and pinched flow (Pødenphant et al. 2015).

Most cell separations were performed in a Newtonian fluid, such as distilled water, or phosphate buffer solution (Lee and Yao 2018; Schaap et al. 2016; Wang et al. 2020) and in non-Newtonian fluid, such as polyethylene oxide and hyaluronic acid (Ahn et al. 2015; Nam et al. 2019; Narayana Iyengar et al. 2021) in inertial straight and spiral microchannels with rectangular and U-shaped cross-sections (Ahn et al. 2015; Mehran et al. 2021; Yuan et al. 2017). Among the passive cell separation methods, inertial microfluidics is important because (i) it can be easily manufactured, (ii) it produces a high yield of separation (Zhang et al. 2018), and (iii) cell separation is solely dependent on hydrodynamic forces such as inertial lift and Dean drag forces which are being generated during fluid flow in the microchannel (Di Carlo et al. 2007). In past studies, (Yuan et al. 2019) used a straight inertial microfluidics device with a rectangular cross-section to separate microalgae cells from bacteria cells in the presence of a polyethylene oxide solution, while (Schaap et al. 2016) used a spiral inertial microfluidics device with rectangular cross-section to separate microalgae of different sizes in distilled water. Moreover (Mehran et al. 2021) used a spiral microchip with a U-shaped cross-section to separate white blood cells from whole blood in distilled water.

This study aimed to fabricate a U- and W- shaped cross-section spiral microchip to separate microalgae from bacteria in the presence/absence of a chemoattractant. We demonstrated that the spiral microchips with U- and W- shaped cross-section structure can be fabricated easily using defocused CO_2_ laser ablation method. To the best of the author’s knowledge, this is the first study to isolate microalgae using a W-shaped cross-section spiral microchip.

## Design principle

Under general conditions, the ratio of inertial force to viscous force (Reynolds number (Re)) is exceptionally small in microfluidic devices (Lee and Yao 2018). However, when the Reynold number is fixed, particles traveling across the spiral microchannel are affected by inertial lift movement effect and Dean flow (Bhagat et al. 2008). The Reynold number of fluids flowing in a spiral microchannel is given in Eq. 1.

1 $$Re=\frac{\rho {U}_{f} {D}_{h}}{\mu }$$

While, particle Reynold number (Rep) is given in Eq. 2

2 $$Re p=\frac{\rho {U}_{f} ap}{\mu }$$

where *ρ* is density of fluid medium (kg/m^3^), ${U}_{f}$ is average fluid velocity (m/s), $\mu$ is represented as the fluid viscosity (kg^−1^ s^−1^), ${D}_{h}=\frac{2\pi {r}^{2}}{(\pi r+d)}$ is the hydraulic diameter of the U-shaped and- W shaped channels (r is the radius and d is the diameter of the microchannel).

The channel curvature causes two counter-rotating vortices (Dean vortices) to be continuous. Hence, the drag forces generated by Dean vortices drive particles to move toward the vortices and the mainstream flow (Al-Halhouli et al. 2018). A dimensionless Dean number can express Dean's vortex strength. The dimensionless number De is given in Eq. 3.

3 $$De=Re\sqrt{ \frac{{D}_{h}}{2R}}$$

**Fig. 1:**
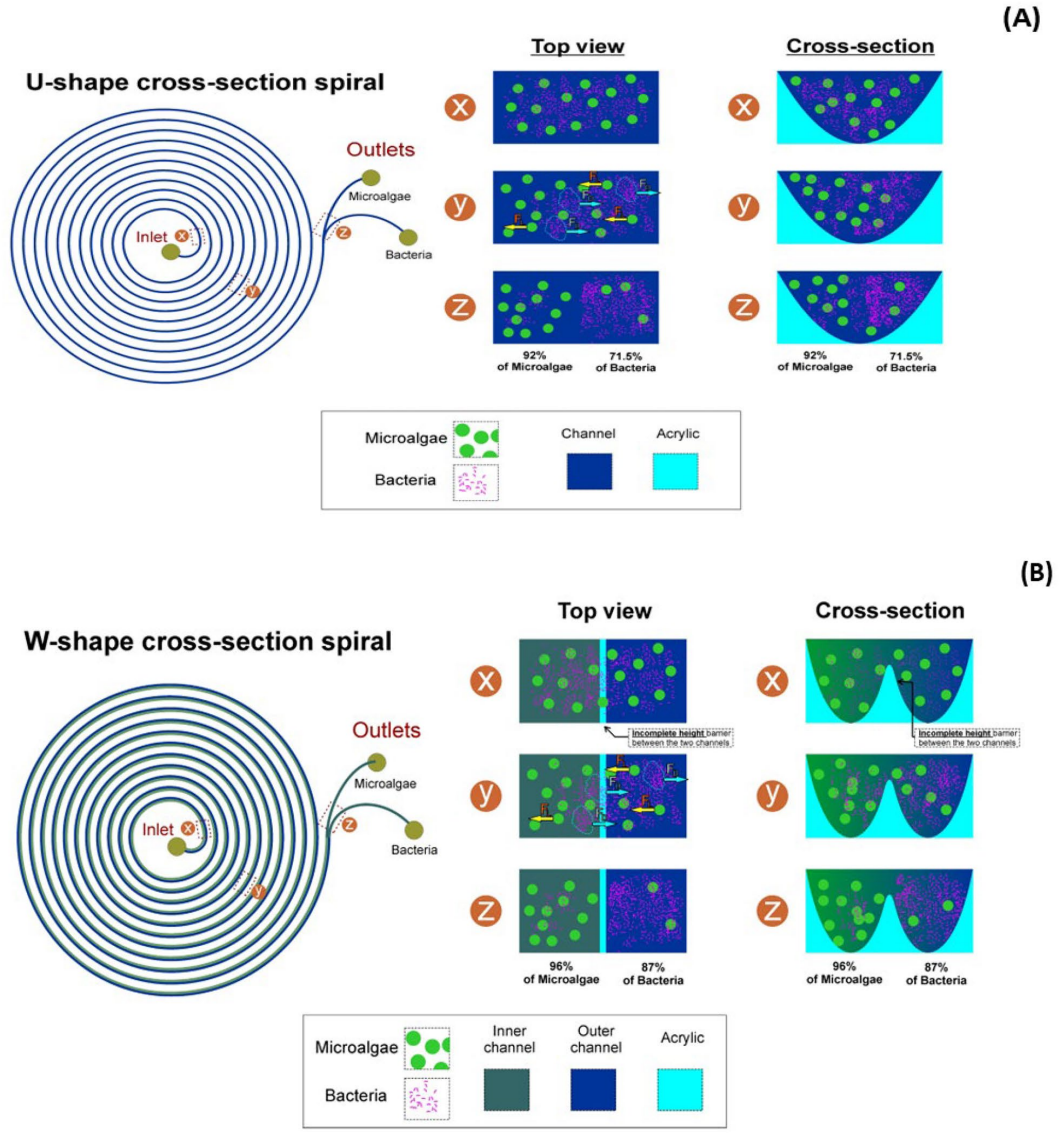
**A**, **B** Show the principle of cell movement in U-shaped and W-shaped cross-section, respectively. **X** represents both microalgae and bacteria cell inside the microchip before separation. **Y** represents the movement of cells in the microchannels under the influence of the inertial lift force acting on larger cells (microalgae) and Dean drag force acting on smaller cells (bacteria). **Z** represents cells at their target outlet with microalgae cells occupying the inner outlet and bacteria cell occupying the outer outlet

*De* is denoted as the Dean number, ${D}_{h}$ is hydraulic diameter, and *R* is radius of curvature. As particles travel along a curved channel due to transverse Dean flow, a drag force is exerted on the particles (Kemna et al. 2012), which moves them towards the Dean vortices (circulate) depending on the particle size to focus either in the inner or outer channel wall (Fig. 1). The Dean drag force is given in Eq. 4.

4 $${F}_{D}= 5.4 \times {10}^{-4}\pi \mu {De}^{1.63}ap$$

The curved geometry of spiral channels generates a force known as inertial force (FL), which affects the flowing particles by focusing the particles to the inner outlet (Fig. 1). FL, which behaves in an inverse direction to Dean drag force, is the balance of the shear gradient lift force (FLS) and wall-induced lift force (FLw) (Al-Halhouli et al. 2018). The inertial lift force is given in Eq. 5.

5 $${F}_{L} =\rho {G}_{2}{C}_{L}a{p}^{4}$$

where *G* is denoted as the fluid shear rate, ${C}_{L}$ is represented as the lift Coefficient 0.5, and *ap* is the particle size.

## Materials and methods

### Design and fabrication of spiral microchannel

**Fig. 2:**
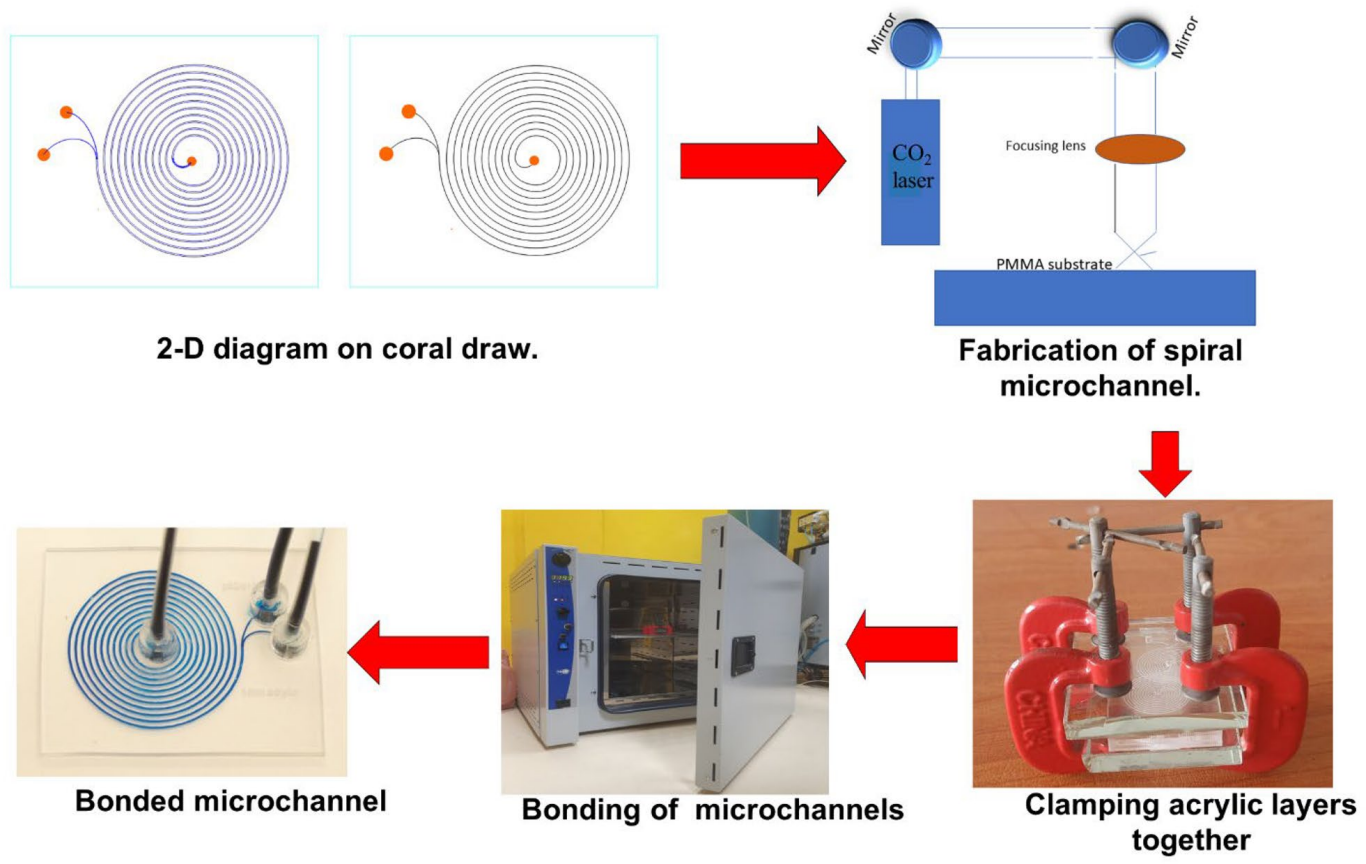
Fabrication steps of spiral microchip

U- and W- shaped spiral microchannels were built in a 2-D form in a coral draw graphics suite X52010 (Fig. 2). In the fabrication of the microchannels, polymethyl methacrylate material (PMMA) cast sheets of 1 mm thickness were used as the microfluidic chip substrate processed by a commercial CO_2_ laser processing machine (VLS3.5 universal laser system Inc. USA). PMMA material was selected because of its biocompatibility and low cost (Helmy et al. 2018; Mehran et al. 2021). In addition, the CO_2_ laser machine was used because of its quick processing time during fabrication (Adel et al. 2023). The laser used in this study has a wavelength of 10.6 μm in the infrared red region, a frequency of 50 KHz, a maximum power of 30 W (Mansour et al. 2022; Nasser et al. 2019a, b; Okello et al. 2022) and a top scan speed of 250 mm/s. During fabrication, the CO_2_ laser cut uses a high-power density focusing optics-lens (HPDFO) of 2 mm focal length with the minimum engraving spot diameter of 100 µm to focus the laser beam on 1 mm acrylic mounted on a working table. The working table was fixed at 3 mm defocused distance. The laser was irradiated using an engraving power of 50% (15 W) and a laser engraving speed of 100% (250 mm/s) (Fig. 2). Hence, the specified spiral microchannel pattern was created based on the laser's output power, constant travel speed, and vector engraving mode, which was set by the coral draw software.

On another 1 mm PMMA substrate, inlet and outlet holes of 2 mm were bored using CO_2_ laser ablation to give fluid access to the microchannel. The fabricated microchannels were washed with distilled water for 10 min and dried using nitrogen gas. The microchannel's depth, width, and cross-section were measured with a 3D laser microscope system (Keyence VK-X100, Keyence Corporation, Osaka, Japan).

Thermal bonding was performed to join the upper and lower PMMA layers together (the top layer containing the inlet and outlets and the bottom layer containing the microchannels) at a temperature of 130 °C for 10 minutes (Fig. 2). Silicon tubes were fitted onto the channel inlet and outlet, and the fabricated microchannels were tested with methylene blue dye (Fig. 2) to inspect for leakages in the microchannels.

### Cell culture

This study employed *Desmodesmus* non-motile, single green algae of ≈15 µm size. These microalgae cells were cultured into a 250 ml conical flask capped with cotton wool containing BG-11 medium for two weeks at room temperature under light conditions. The grown microalgae were transferred into a 15 ml falcon tube under aseptic conditions and centrifuged for 10 minutes at 4000 rpm, and the pellet was resuspended in distilled water.

*Escherichia coli* (*E. coli*) is a motile, rod-shaped bacteria with an average diameter of about 1µm. Bacteria were cultured in a shaker in Luria Bertani broth (2 g/ml NaCl, 2 g/ml tryptone, 1 g/ml yeast extract into 200 ml distilled water) for 48 hrs. The overnight growth of cultured bacteria was measured using a spectrophotometer (EMCLAM instruments, Germany) at Optical density (O.D_600_). The grown bacteria were centrifuged at 4000 rpm, and the bacteria pellet was resuspended in distilled water and kept at room temperature.

### Cell count

Microalgae were counted using a haemocytometer (Paul Marienfeld Counting Chamber, Germany) in the four large grid squares, and a microalgae concentration $\mathrm{of }6000\mathrm{ cells}/{\text{ml}}$ was acquired.

### Experimental setup

**Fig. 3:**
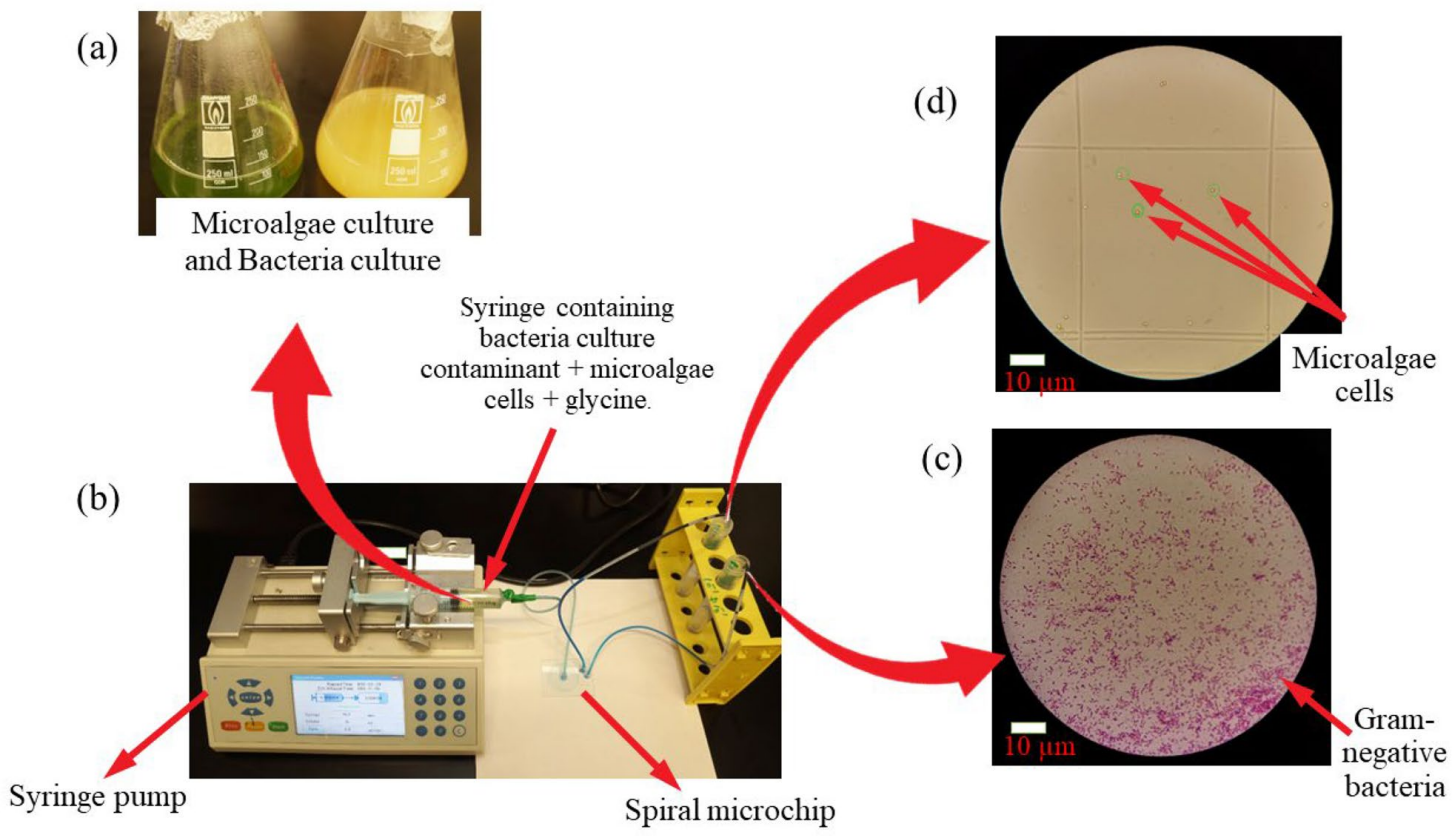
Experimental setup **A** microalgae growth in BG11 media and bacteria cells growth in Luria Bertani agar, **B** 10 ml plastic syringe with mixtures of cells in glycine been pumped into the spiral microchip using a syringe pump, **C** micrograph of bacteria cells collected at the outer outlet after separation and gram staining **D** micrograph of microalgae cells collected at inner outlet and counted using a hemocytometer

Two ml of bacterial culture were mixed with 2 ml of microalgae culture with and without glycine and injected into the microchannels using a 10 ml plastic syringe supported onto a syringe pump (Chemyx Fusion 200, USA), as illustrated in Fig. 3. In these spiral microchips, experiments were conducted at a flow rate ranging from 0.3 ml/min to 0.9 ml/min. Bacteria cells were collected at the outer outlet of the microchannels after cell separation and grown on Luria Bertani agar to count the number of bacteria cells recovered at the outer outlet at each flow rate. Meanwhile, microalgae were collected from the inner outlet and the number of microalgae obtained at each flow rate was counted using a hemocytometer.

This study defined microalgae separation efficiency as the number of microalgae obtained at the inner outlet (target outlet) to the total number of microalgae in the inner and outer outlet. Equation 6 shows the formula to calculate microalgae separation efficiency.

6 $${E}_{m}=\frac{{N}_{io}}{ {N }_{(io+oo) }}\times 100$$

where ${E}_{m}$ is the microalgae separation efficiency, ${N}_{i0}$ is the number of microalgae at the inner outlet (microalgae target outlet) and ${N}_{(io+oo)}$ is the number of microalgae at the inner and outer outlets. The bacterial removal ratio was defined as the number of bacteria cells obtained at the outer outlet (bacteria target outlet) to the total number of cells in the outer and inner outlet. Bacteria removal ratio was calculated using Eq. 7.

7 $${E}_{b}= \frac{{N}_{oo}}{ {N }_{(oo+io) }}\times 100$$

where ${E}_{b}$ is the bacterial removal ratio, $${N}_{\begin{array}{c}oo\\ \end{array}}$$ is the total number of bacteria at the outer outlet (target outlet) and ${N}_{(oo+io)}$ is the total number of bacteria at the outer and inner outlets.

## Results and discussion

### Microchannel characterization

This study used the defocusing CO_2_ laser ablation method to fabricate spiral microchips with U- and W-shaped cross-sections. The measured width of the U-shaped cross-section was 227 µm while the measured depth ranged from 175 to 210 µm. The width of 190 µm was used as a middle value to calculate the hydraulic diameter of the microchannel. The measured width of W- shaped cross-section was 220 µm while the measured depth ranged from 162 to 210 µm, and the width of 180 µm was used as the middle value to calculate the hydraulic diameter of the microchannel.

**Fig. 4:**
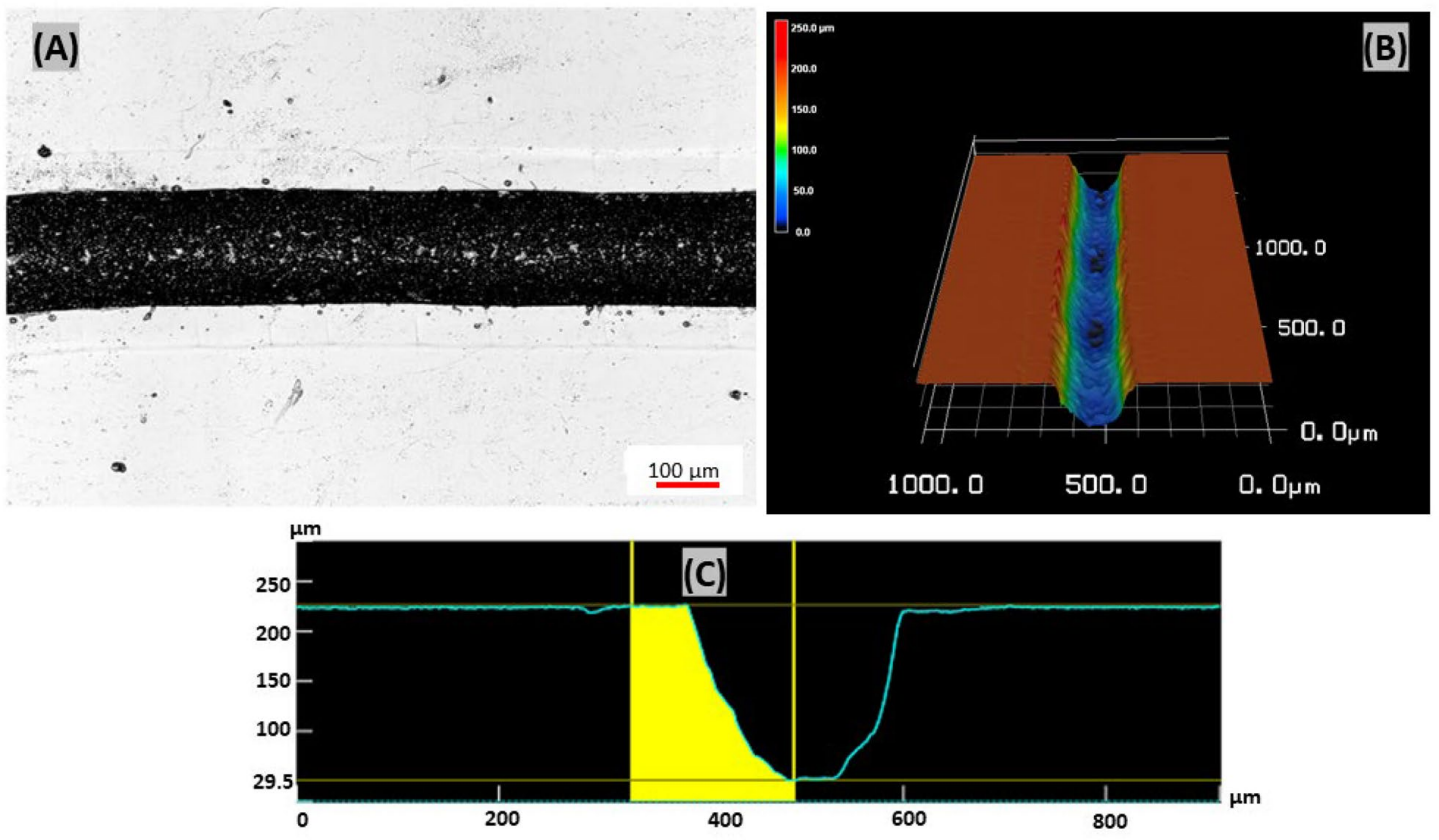
Microchannel fabricated on PMMA material using defocused laser ablation method: **A** Optical channel image, **B** 3D micrograph of the single spiral microchannel and **C** U-shaped cross-section channel profile

The single spiral microchannel fabricated by a single laser scan has a U-shaped cross-section while the double-shaped spiral microchannel fabricated by the double scan method with slightly displaced second of the laser has a W-shaped cross-section. In a U- shaped cross-section, when the laser beam strikes an acrylic material surface, the laser beam engraves a single spiral microchannel on the acrylic material surface at a distance of 3 mm from the focal point, which causes the energy from the laser beam to spread across a larger region producing U-shaped cross-section spiral microchannels with shallower depth and wider width (Fig. 4).

**Fig. 5:**
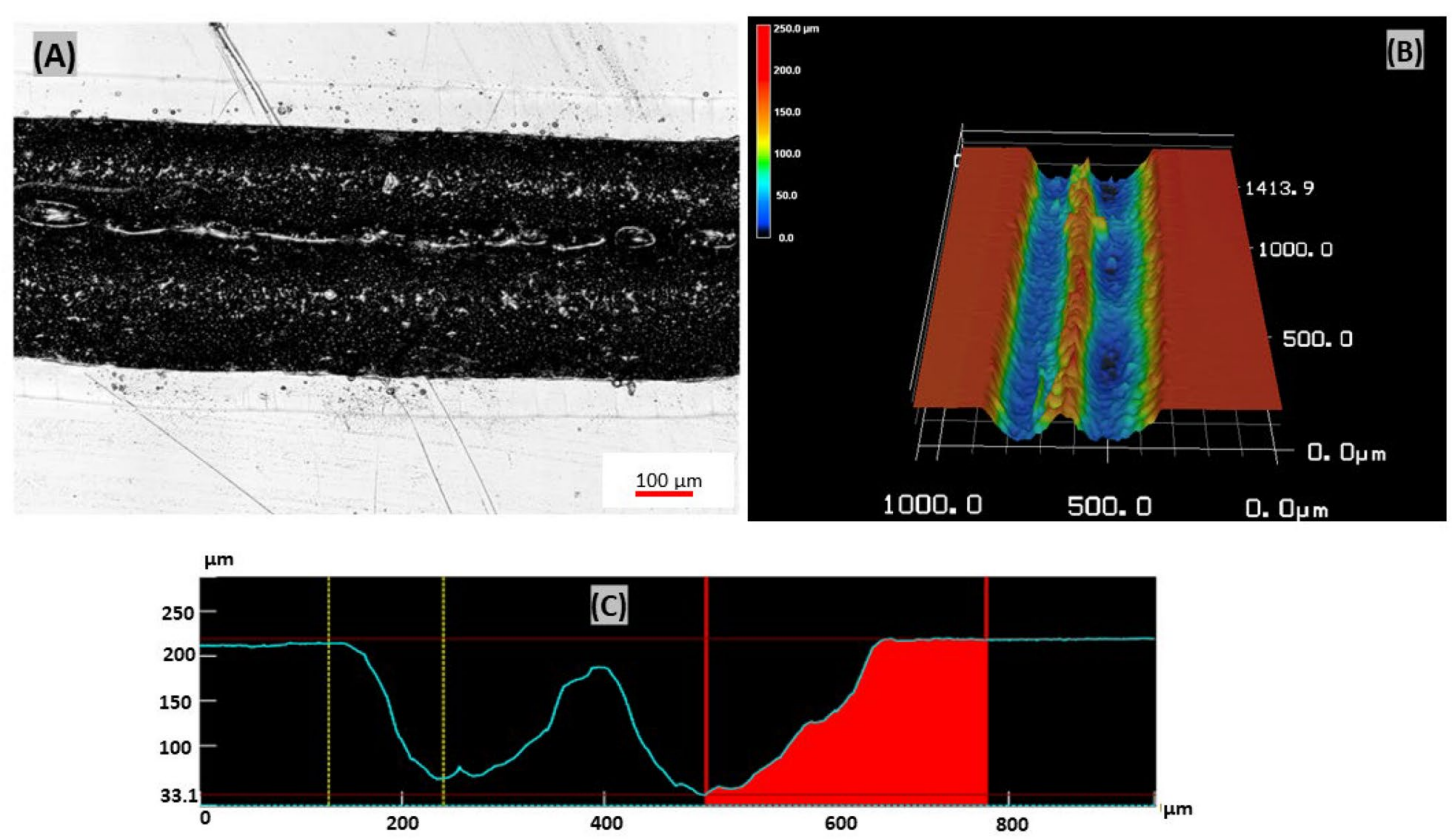
**A** Optical channel image, **B** 3D micrograph of double spiral microchannel and **C** W-shaped cross-section channel profile

Meanwhile, in spiral microchannel with W-shaped cross-section, when the laser beam strikes the surface of an acrylic material the laser beam engraves two spiral microchannels along with two slightly displaced paths, having a 100 µm distance between every two spirals on the acrylic material at a distance of 3 mm from the focal point, which causes the energy from the laser beam to spread across a larger region on the acrylic material resulting in two spiral microchannels intersecting together thereby forming an incomplete barrier between the two channels leading to a W- shaped cross-section spiral microchannel (Fig. 5).

**Fig. 6:**
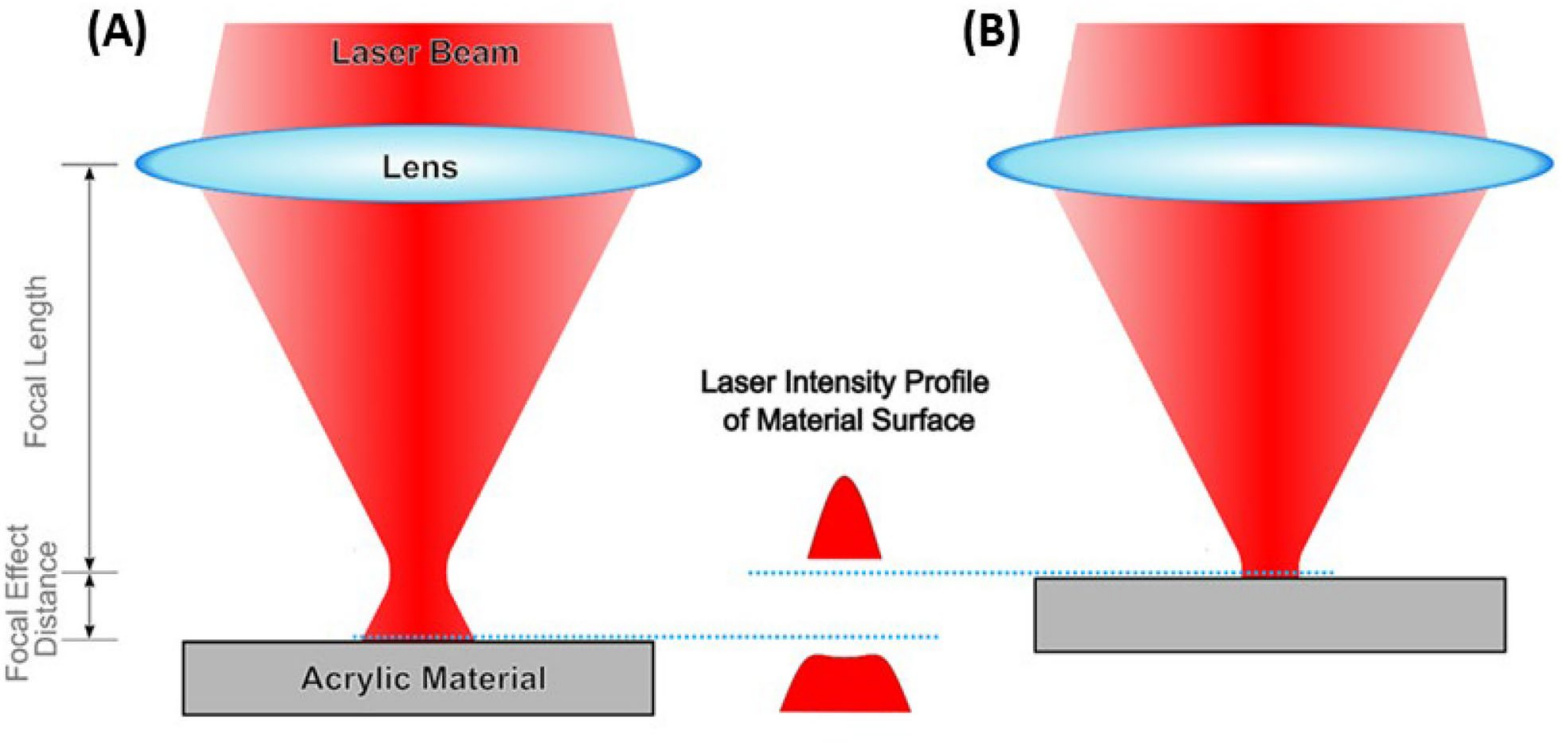
**A** The defocused method of the CO_2_ laser machine with a 3 mm defocusing distance away from the material forms microchannels. **B** The focused method of the CO_2_ laser machine with direct focusing of the laser beam onto the acrylic material forms a Gaussian shape cross-section

In contrast to the focusing method which produces Gaussian-shaped microchannels with deeper depth and narrower width when the laser beam strikes the acrylic material at a distance of 0 mm from the focal point, the defocused method (used in this study) produces microchannels with wider width and shallower depths (Nasser et al. 2018) (Fig. 6).

#### Effect of glycine on cell separation

This study investigated the effect of the presence and absence of glycine (200 µM) on the separation of microalgae from bacterial contaminants. The 200* µ*M was used because the EC_50_ of *E. coli* was found to be above 100* µ*M (Yang et al. 2015). During bacteria movement in the microchannels, when glycine bound to the Tar or Tas receptor of *E*. *coli*, glycine served as a source of nutrients by auto-phosphorylating a histidine kinase CheA. The phosphate group in this phosphorylated CheA was transferred to the response regulator CheY. This phosphorylated CheY bound to the flagellar motors of the *E. coli,* thus favored the movement of the *E. coli* towards their outer outlet. As a result, Dean drag force and glycine acted on bacteria cells, led to high removal of bacterial cells in the U- and W-shaped cross-section spiral microchannels of 72% and 87%, respectively. In a study by (Yuan et al. 2019), only elastic forces brought by polyethylene oxide solution acted on the bacteria cell, thus increasing bacterial movement towards the outer outlet, leading to a high bacteria removal ratio of 92.97%. In this study, when the experiment was performed without glycine, a low bacterial removal ratio of 63% and 66% was obtained using U- and W-shaped cross sections, respectively. In this case, the removal ratio is achieved only by Dean drag force. Microalgae cells lack flagella that bind with glycine. This indicates that the movement of microalgae in the presence and absence of glycine depends on inertial lift forces.

### Effect of Reynold number and Dean number on fluid flow in microchannel

Equations 1 and 3 above were used to calculate the Reynold number and Dean number of fluid flow in the microchannel, which would be used to predict the type of flow experienced by the fluid in the microchannels (Table 1). The density of glycine in water was calculated to be 948.81 kg/m^3^ using the formula $\frac{M}{V} .$ Where *M* = glycine solution mass and V is glycine solution volume, while the density of water was 1000 kg/m^3^. Equation 2 above was used to calculate the particle Reynold number (Table 2).

**Table 1:** Calculated Re and De numbers at different flow rates.

Flow rates (ml/min)	W-shaped				U-shaped			
	Water		Glycine		Water		Glycine	
	Re	De	Re	De	Re	De	Re	De
0.3	81.5	11.4	84.2	11.8	36.6	5.10	35.4	4.10
0.4	109	15.2	112	15.7	47.6	6.60	46.1	6.50
0.5	136	19.0	140	19.6	58.6	8.20	56.6	7.90
0.6	164	22.1	170	23.8	69.5	9.70	67.3	9.40
0.7	190	26.6	196	27.4	82.1	11.6	80.3	11.2
0.8	219	30.6	226	31.6	95.2	13.3	92.3	12.9
0.9	236	33.1	244	34.2	107	15.0	104	14.6

**Table 2:** Rep of microalgae cells (15 µm) and bacteria cells (1 µm) for the W- and U-shape

Flow rates (ml/min)	W-shape		U-shaped	
	Rep (15 µm)	Rep (1 µm)	Rep (15 µm)	Rep (1 µm)
0.3	4.80	0.32	4.50	0.30
0.4	6.50	0.43	5.90	0.39
0.5	7.10	0.53	7.20	0.48
0.6	10.0	0.64	8.60	0.57
0.7	11.1	0.74	10.2	0.68
0.8	12.7	0.85	11.7	0.78
0.9	14.4	0.96	13.2	0.88

In the microchannels, the fluid experienced a laminar flow both in glycine solution and distilled water causing the movement of fluid to be in a smooth and orderly manner in the microchannels since their Reynold number (Re) is less than 2000. The Dean number (De) indicates that, the curvature of the spiral microchip brings about secondary force in the microchannel known as Dean drag forces which act on smaller cells.

### Effect of flow rate on cell separation: microalgae separation efficiency

**Fig. 7:**
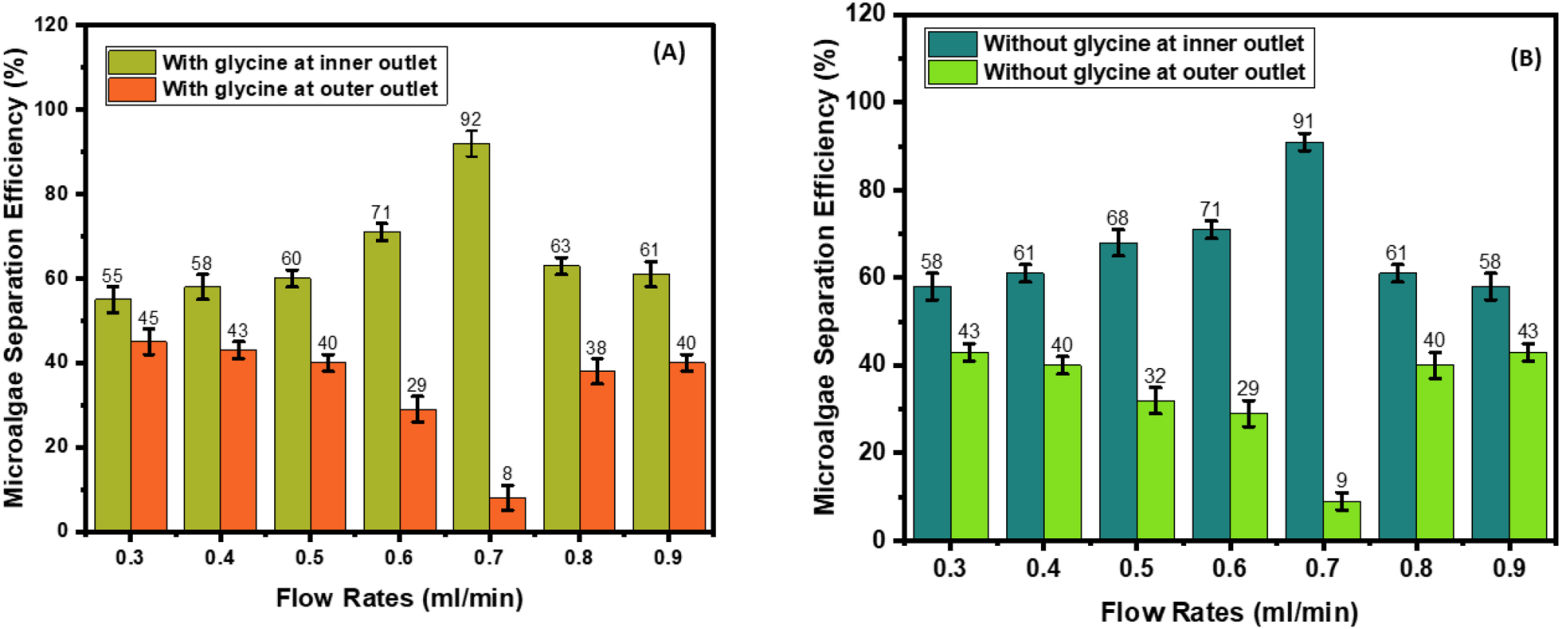
Microalgae separation efficiency in U-shaped cross-section spiral microchip. **A** with and **B** without glycine

In the U-shaped cross-section spiral microchip, in the presence of glycine, as the flow rate increases from 0.3 ml/min to 0.7 ml/min, microalgae separation efficiency increases from 55% to 92%. As the flow rate increases from 0.8 to 0.9 ml/min, microalgae separation efficiency decreases from 63% to 61% (Fig. 7A). In the absence of glycine, as the flow rate increases from 0.3 ml/min to 0.7 ml/min, microalgae separation efficiency from bacteria contaminant increases from 58% to 91%. As the flow rate increases from 0.8 to 0.9 ml/min, microalgae separation efficiency decreases from 61 to 58%, respectively (Fig. 7B).

**Fig. 8:**
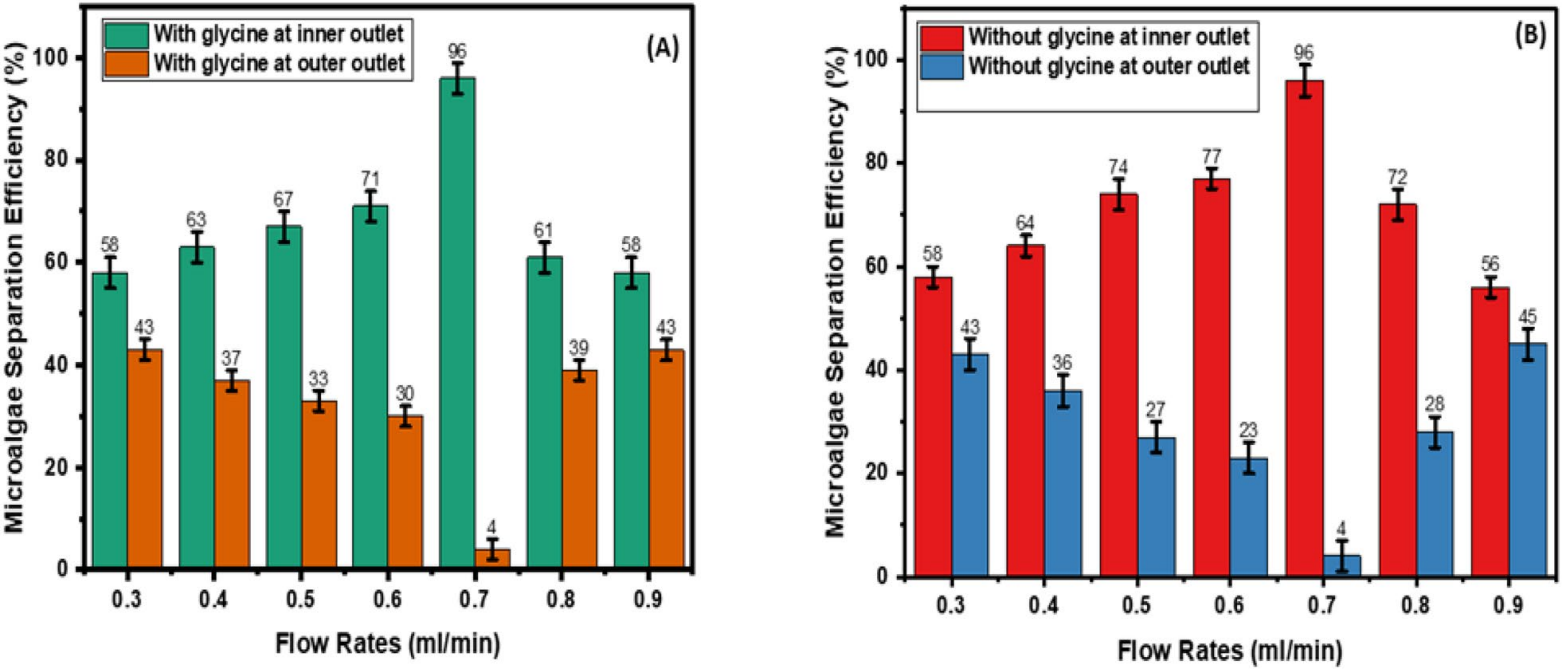
Microalgae separation efficiency in W-shaped cross-section spiral microchip. **A** with and **B** without glycine

In the W-shaped cross-section, in the presence of glycine, as the flow rate increases from 0.3 ml/min to 0.7 ml/min, microalgae separation efficiency increases from 58% to 96%. As the flow rate increases from 0.8 ml/min to 0.9 ml/min, microalgae separation efficiency decreases from 61 to 58%, respectively (Fig. 8A). In the absence of glycine, as the flow rate increases from 0.3 ml/min to 0.7 ml/min, microalgae separation efficiency increases from 58 to 96%. As the flow rate increases from 0.8 ml/min to 0.9 ml/min, microalgae separation efficiency decreases from 72 to 56%, respectively (Fig. 8B).

This high separation efficiency of microalgae obtained in both U- and W- cross-section (as the flow rate increases from 0.3 ml/min to 0.7 ml/min) is because microalgae has: (i) a blockage ratio (ß) of 0.1 (Yuan et al. 2019), (ii) a high channel and particle Reynold number (Tables 1 and 2 respectively) (Schaap et al. 2016), and (iii) an inertial ratio of 0.33 and 0.37 (*R*_*f*_ = $\frac{a{p}^{2}R}{D{h}^{3}}$> 0.04) (Kemna et al. 2012), causing inertial lift forces to dominate the movement of the microalgae in the microchannel. In contrast to our study, a study by (Schaap et al. 2016), found out that *Monoraphidium* microalgae requires a higher flow rate of more than 1 ml/min to achieve their focusing position in the microchannel due to their small size (3 µm), a low *R*_*f*_ < 0.04 in their experiment and the action of smaller lift forces on *Monoraphidium*.

A low microalgae separation efficiency was obtained as the flow rate increased from 0.8 ml/min to 0.9 ml/min because of an increase in Dean vortex strength (Table 1), which causes the cells to remain dispersed around the microchannel. As such, Dean drag forces dominate the movement of microalgae, causing the microalgae to move away from their initial focusing positions at the inner outlet and concentrate towards the outer outlet (Mehran et al. 2021). This is in accordance with a study by (Syed et al. 2018), who stated that as the flow rate increases to 1.75 ml/min, the separation efficiency of microalgae *T. suecica* decreases to 8% due to a rise in Dean drag forces over lift forces.

### Bacterial removal ratio

**Fig. 9:**
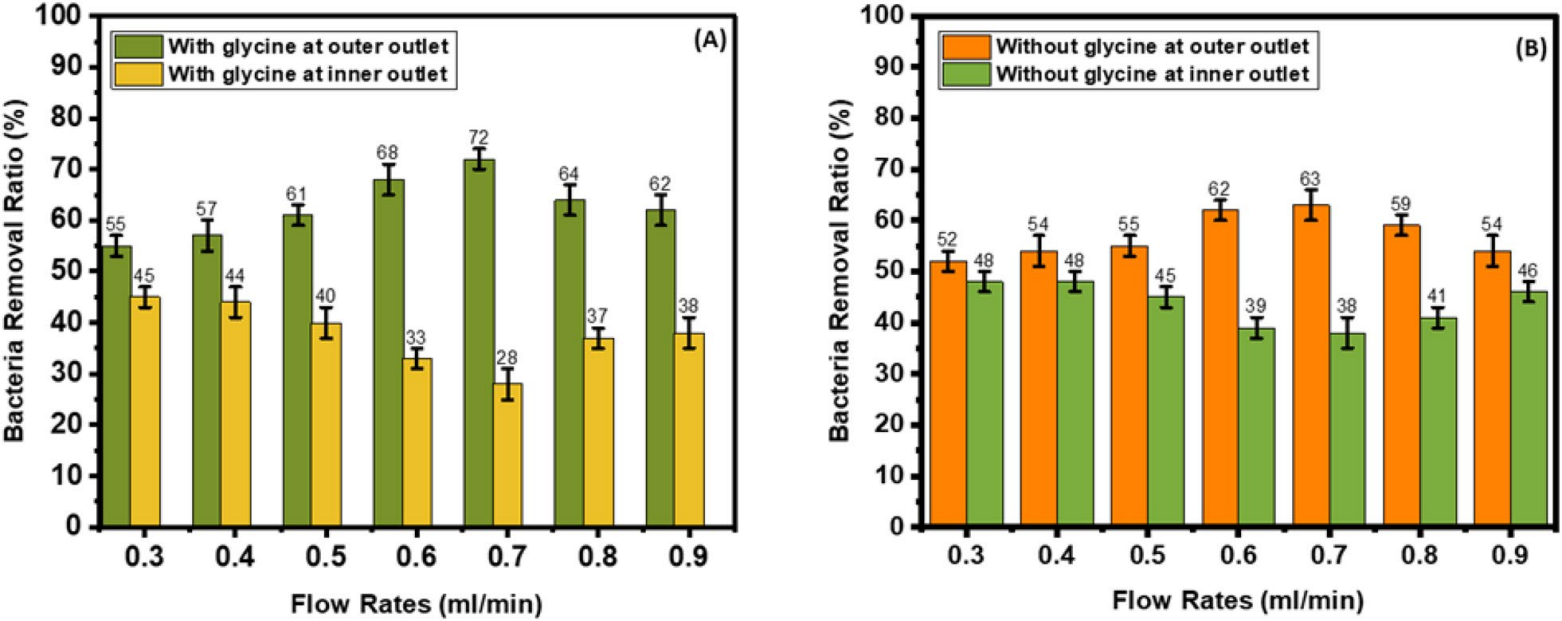
Bacteria removal ratio in U-shaped cross-section. **A** With and **B** without glycine

**Fig. 10:**
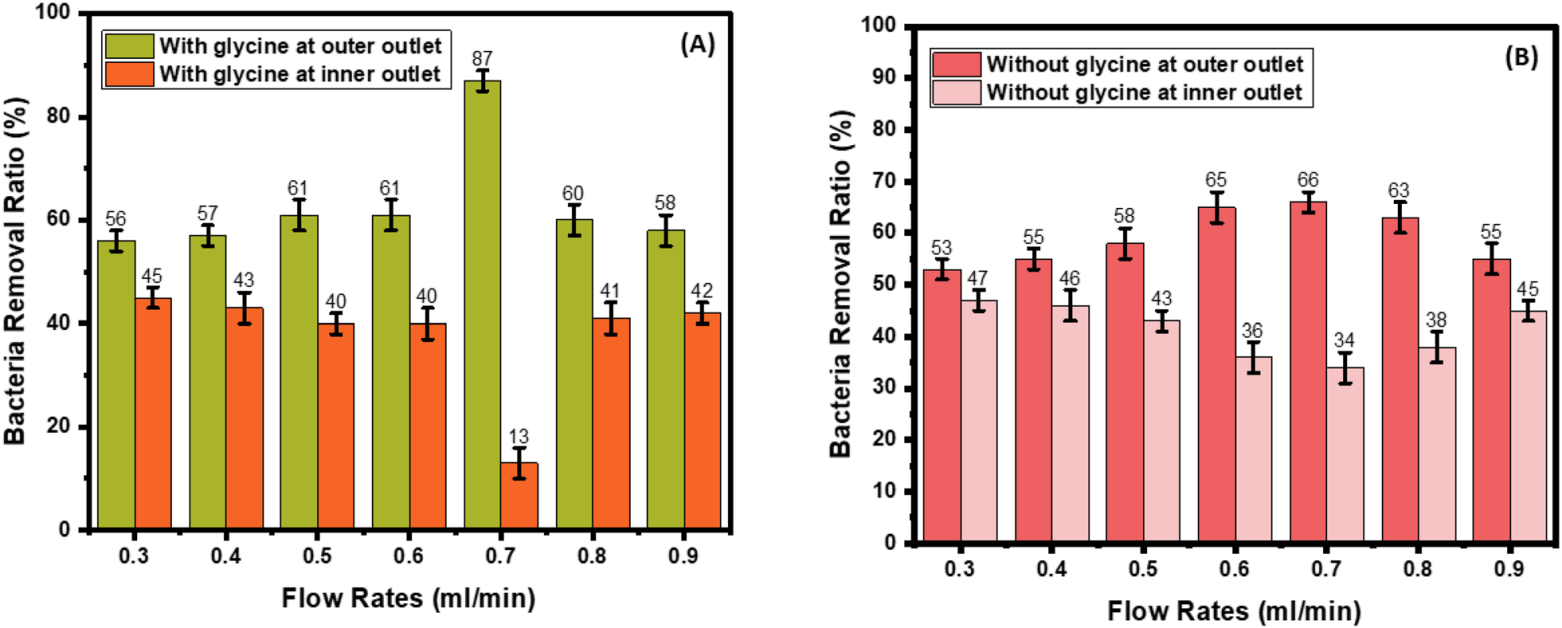
Bacteria removal ratio in W-shaped cross-section. **A** With and **B** without glycine

In the U-shaped cross-section in the presence of glycine, as the flow rate increases from 0.3 ml/min to 0.7 ml/min, the bacteria removal ratio increases from 55 to 72%. As the flow rate increases from 0.8 ml/min to 0.9 ml/min, the bacteria removal ratio decreases from 64 to 62% (Fig. 9A). In the absence of glycine, as the flow rate increases from 0.3 ml/min to 0.7 ml/min, the bacteria removal ratio increases from 52 to 63%. As the flow rate increases from 0.8 ml/min to 0.9 ml/min, the bacteria removal ratio decreases from 59 to 54% (Fig. 9B). However, in the W-shaped cross-section, as the flow rates increase from 0.3 ml/min to 0.7 ml/min, the bacteria removal ratio increases from 56 to 87%. As the flow rate increases from 0.8 ml/min to 0.9 ml/min, the bacteria removal ratio decreases from 60 to 58% (Fig. 10A). In the absence of glycine, as the flow rate increases from 0.3 ml/min to 0.7 ml/min, the bacteria removal ratio increases from 53 to 66%. As the flow rate increases from 0.8 to 0.9 ml/min, the bacteria removal ratio decreases from 63 to 55% (Fig. 10B).

Generally, at the flow rates from 0.3–0.9 ml/min, most bacteria cells remain dispersed in W- and U-shaped cross-section. This is because the average size of *E. coli* is 1 µm which do not obey the size criteria for inertial focusing due to (i) a low bacteria blockage ratio (ß) of 0.008 (Mehran et al. 2021; Yuan et al. 2019) (ii) a hydraulic diameter (*D*_*h*_*)* of 1.3 × 10^2^, 1.2 × 10^2^, (iii) a high channel Reynold number and a low particle Reynold number (Tables 1 and 2) (Schaap et al. 2016) and (iv) a low inertial ratio (*R*_*f*_) < 0.04 (Kemna et al. 2012) in U-shaped and W shaped cross-section microchips which affect the focusing ability of bacteria in the microchannel there by reducing their removal ratio at their target outlet. This is in accordance with a study by Schaap et al. (2016), where most microalgae *Chlorella* cells of 5 µm in size did not focus in the spiral microchip because a microchannel depth of 100 µm was used. A decrease in the microchannel depth from 100 to 25 µm and an increase in flow rate to more than 1.4 ml/min, favored the focusing of *Chlorella* cells in a narrow stream with higher separation efficiency of more than 90%. Whereas in another study by (Yuan et al. 2019), most of the bacteria cells were not dispersed in the microchannel because the microchannel had a depth of 10 µm, which favored the movement and focusing of bacteria *(Bacillus subtilis)* at the outer outlet of the microchannel, leading to a high removal ratio of 92.97%.

**Fig. 11:**
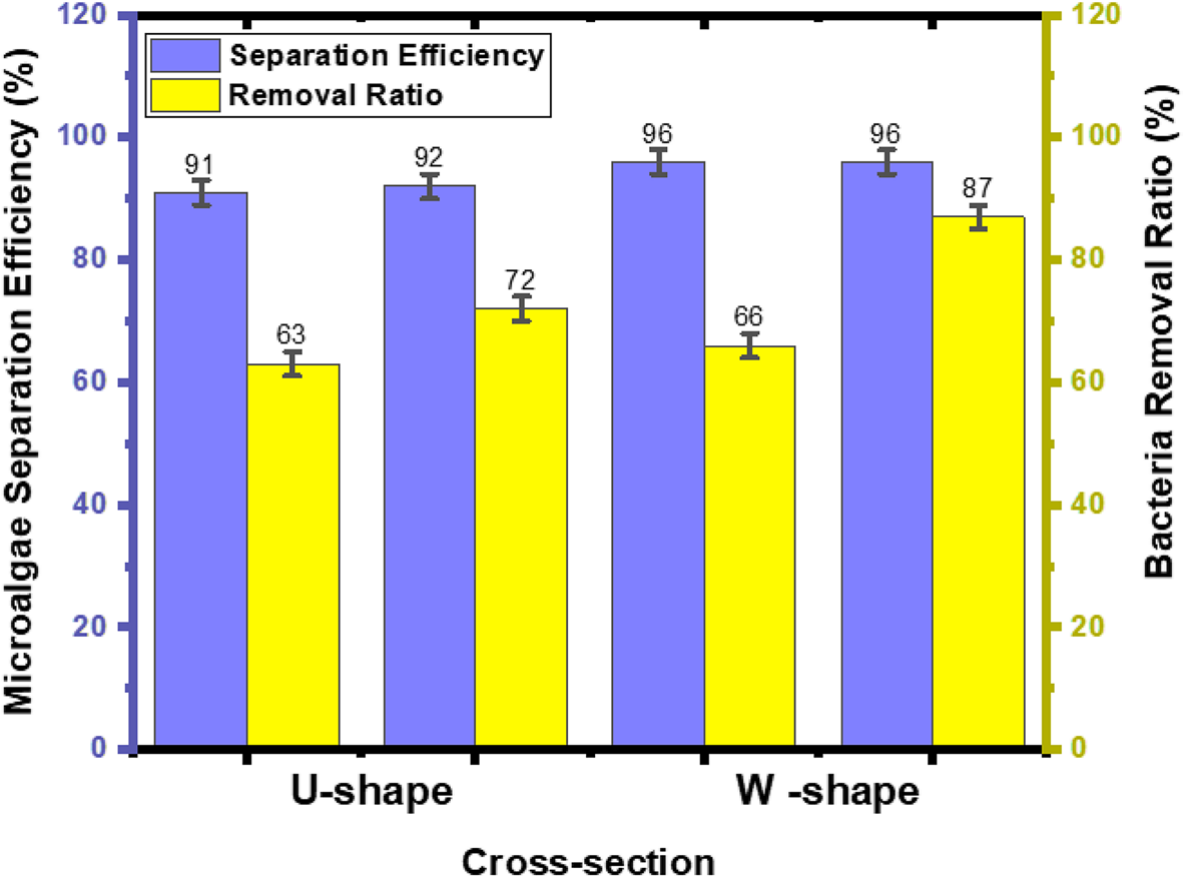
A comparison of microalgae separation efficiency and bacteria removal in U and W-shaped cross sections in the presence and absence of glycine

The W-shaped cross-section produces better microalgae (*Desmodesmus* sp) separation efficiency and better bacteria removal ratio in the presence and absence of glycine (Fig. 11) because a barrier created at the center of the microchannels prevents some recirculation and mixing of microalgae and bacteria cells in the microchannel. This is in contrasts to the U-shaped cross-section, which produces low microalgae (*Desmodesmus* sp*)* separation efficiency and low bacteria *(E. coli)* removal ratio both in the presence and absence of glycine. In another study utilizing a spiral microfluidics device with a rectangular cross-section produced a 60–80% separation efficiency of *Cosmarium* from *Chlorella vulgaris* (Lee and Yao 2018)*.* Moreover, a study by (Wang et al. 2020) using spiral devices with rectangular cross-sections reported more than 80% *Chlorella* separation efficiency from *Closterium* and *Platymonas.* The low microalgae separation efficiencies and bacteria removal ratio in spiral microchips with U-shaped and rectangular-shaped cross sections was due to the absence of a barrier at the centre of the spiral microchannels, which thus causes recirculation and mixing cells to be a prevalent problem in these microchips.

A summary of previous studies assessing the effects of microchannel cross-sections and fluid medium on the efficiency of microalgae separation and bacteria removal ratio is presented in Table 3.

**Table 3:** Summary of previous studies in comparison to this study

Studies	Fluid medium	Cross-section	Microalgae Separation eff. (%)	Bacteria removal ratio (%)	References
*Desmodesmus* sp and *E. coli*	Glycine solution	W-cross-section	96	87	This study
*Desmodesmus* sp and *E. coli*	Distilled water	W- cross-section	96	66	This study
*Desmodesmus* sp and *E. coli*	Glycine solution	U-cross-section	92	72	This study
*Desmodesmus* sp and *E. coli*	Distilled water	U-cross- section	91	63	This study
Chlorella vulgaris and Bacillus subtilis	Polyethylene oxide	Rectangular cross-section	100	92.97	Yuan et al. (2019)
*Cosamarium* and* Chlorella vulgaris*	Distilled water	Rectangular cross-section	60–80	–	Lee and Yao (2018)
*Chlorella* from *Closterium* and *Platymonas*	Phosphate buffer solution	Rectangular cross-section	> 80	–	Wang et al. (2020)
*Monoraphidium* and *Cyanothece*	Distilled water	Rectangular cross-section	77	–	Schaap et al. (2016)
*Platymonas *and *Closterium*	Phosphate buffer solution	Rectangular cross -section	90	–	Wang et al. (2019)
*Tetraselmis* sp and *Chlorella* sp	Distilled water	Rectangular cross-section	≥ 90	–	Wang et al. (2021)
C. *reinhardtti* and *E. coli*	Distilled water	Rectangular cross-section	100	100	Robla et al. (2021)
*B. subtilis* with varying sizes	Polyethylene oxide solution	Rectangular cross-section	97.5		Liu et al. (2022)
Rhodomonas, *Alexandrum* and *E. coli*	Distilled water	Circular cross-section	89	75	Korensky et al. (2021)
*Platymonas* and *Chlorella*	Phosphate buffer solution	Triangular cross-section	85.7	–	Wang (2021)

In the rectangular cross-section, there were variations in the percentage of microalgae separation efficiency according to the fluid medium used. In the case of using distilled water as fluid medium, the separation efficiency varied from 60 to 100% (Schaap et al. 2016; Robla et al. 2021; Wang et al. 2022; Lee and Yao 2018). The 100% separation efficiency obtained in the study by (Robla et al 2021) was because of using a nonporous membrane as sandwiched between the microfluidics channels during cell separation. The use of polyethylene oxide solution as a fluid medium, resulted in a higher separation efficiency of 97.5 to 100% (Liu et al. 2022; Yuan et al. 2019). Meanwhile, using phosphate buffer solution in the rectangular cross-section microchip, the efficiency ranged from 80 to 90% (Wang et al. 2019; Wang et al 2020; Wang 2021). The lowest separation efficiency was reported by (Wang et al 2021; Korensky et al. 2021) who used triangular and circular cross-section with phosphate buffer solution and distilled water as fluid medium, respectively. This may conclude that a rectangular cross-section in the presence of a polymer solution (Polyethylene oxide) and in the presence of distilled water, during cell separation provided a high separation efficiency and bacterial removal ratio.

## Conclusion

W- and U-shaped cross-sectional spiral microchips were fabricated by defocusing CO_2_ laser ablation. The U-shaped cross-section showed a microalgae separation efficiency of 92% and a bacteria removal ratio of 72% with glycine, and a 91% microalgae separation efficiency and a bacteria removal ratio of 63% without glycine. The W-shaped cross-section spiral microchip showed a microalgae separation efficiency of 96% and a bacteria removal ratio of 87% with glycine, and 96% microalgae separation efficiency and a bacteria removal ratio of 66% without glycine. The spiral microchips fabricated in this study can be widely applied to other cell separation applications, such as white blood cell separation from whole blood, circulating tumor cell separation from urine, and microalgae separation in droplet-based microfluidics.

For future work, we may need to investigate other fabrication methods such as the soft lithography to obtain U and W-shaped cross section of less than 100 µm depth. Other improvements maybe done by adding a nanoporous membrane between the microchannels before separation, elastic fluids such as xanthan gum, hyaluronic acid, polyethylene oxide solution may be applied during cell separation to increase microalgae separation efficiency. In addition, various glycine concentrations should be tested for separation of various types of motile bacteria.

## Data Availability

All data obtained in this studies are found in this article.

## Abbreviations

Re: Reynold number
Rep: Particle Reynold number
De: Dean number

## References

[CR1] Adel M, Allam A, Sayour AE, Ragai HF, Umezu S, El-bab AMRF (2023). Fabrication of spiral low-cost microchannel with trapezoidal cross section for cell separation using a grayscale approach. Micromachines.

[CR2] Ahn SW, Lee SS, Lee SJ, Kim JM (2015). Microfluidic particle separator utilizing sheathless elasto-inertial focusing. Chem Eng Sci.

[CR3] Al-Halhouli A, Al-Faqheri W, Alhamarneh B, Hecht L, Dietzel A (2018). Spiral microchannels with trapezoidal cross section fabricated by femtosecond laser ablation in glass for the inertial separation of microparticles. Micromachines.

[CR4] Bakhshi MS, Rizwan M, Khan GJ, Duan H, Zhai K (2022). Design of a novel integrated microfluidic chip for continuous separation of circulating tumor cells from peripheral blood cells. Sci Rep.

[CR5] Bhagat AAS, Kuntaegowdanahalli SS, Papautsky I (2008). Continuous particle separation in spiral microchannels using dean flows and differential migration. Lab Chip.

[CR6] Bodénès P, Wang HY, Lee TH, Chen HY, Wang CY (2019). Microfluidic techniques for enhancing biofuel and biorefinery industry based on microalgae. Biotechnol Biofuels.

[CR7] Borowitzka MA (2013). High-value products from microalgae-their development and commercialisation. J Appl Phycol.

[CR8] Chung AJ (2019). A minireview on inertial microfluidics fundamentals: inertial particle focusing and secondary flow. BioChip J.

[CR9] Di Carlo D, Irimia D, Tompkins RG, Toner M (2007). Continuous inertial focusing, ordering, and separation of particles in microchannels. Proc Natl Acad Sci USA.

[CR10] Hejazian M, Li W, Nguyen NT (2015). Lab on a chip for continuous-flow magnetic cell separation. Lab Chip.

[CR11] Helmy MO, El-Bab ARF, El-Hofy HA (2018). Fabrication and characterization of polymethyl methacrylate microchannel using dry and underwater CO_2_ laser. Proc Inst Mech Eng.

[CR12] Kang DH, Kim K, Kim YJ (2018). An anti-clogging method for improving the performance and lifespan of blood plasma separation devices in real-time and continuous microfluidic systems. Sci Rep.

[CR13] Kemna EWM, Schoeman RM, Wolbers F, Vermes I, Weitz DA, Van Den Berg A (2012). High-yield cell ordering and deterministic cell-in-droplet encapsulation using Dean flow in a curved microchannel. Lab Chip.

[CR14] Kim GY, Son J, Han JI, Park JK (2021). Inertial microfluidics-based separation of microalgae using a contraction–expansion array microchannel. Micromachines.

[CR15] Korensky G, Chen X, Bao M, Miller A, Lapizco-Encinas B, Park M, Du K (2021). Single *Chlamydomonas reinhardtii* cell separation from bacterial cells and auto-fluorescence tracking with a nanosieve device. Electrophoresis.

[CR16] Lee ML, Yao DJ (2018). The separation of microalgae using Dean flow in a spiral microfluidic device. Inventions.

[CR17] Liu P, Liu H, Semenec L, Yuan D, Yan S, Cain AK, Li M (2022). Length-based separation of *Bacillus subtilis* bacterial populations by viscoelastic microfluidics. Microsyst Nanoeng.

[CR18] Mansour H, Soliman EA, El-Bab AMF, Abdel-Mawgood AL (2022). Development of epoxy resin-based microfluidic devices using CO_2_ laser ablation for DNA amplification point-of-care (POC) applications. Int J Adv Manuf Technol.

[CR19] McGrath J, Jimenez M, Bridle H (2014). Deterministic lateral displacement for particle separation: A review. Lab Chip.

[CR20] Mehran A, Rostami P, Saidi MS, Firoozabadi B, Kashaninejad N (2021). High-throughput, label-free isolation of white blood cells from whole blood using parallel spiral microchannels with u-shaped cross-section. Biosensors.

[CR21] Morales-Jiménez M, Gouveia L, Yañez-Fernandez J, Castro-Muñoz J, Barragan-Huerta BE (2020). Microalgae-based biopolymer as a potential bioactive film. Coatings.

[CR22] Morales-Jiménez M, Yáñez-Fernández J, Castro-Muñoz R, Barragán-Huerta BE, Jafari SM, Castro-Muñoz R (2021). Recovery of High Added Value Compounds from Microalgae Cultivation Using Membrane Technology. Membrane Separation of Food Bioactive Ingredients.

[CR23] Mourelle ML, Gómez CP, Legido JL (2017). The potential use of marine microalgae and cyanobacteria in cosmetics and thalassotherapy. Cosmetics.

[CR24] Nam J, Jee H, Jang WS, Yoon J, Park BG, Lee SJ, Lim CS (2019). Sheathless shape-based separation of candida albicans using a viscoelastic non-newtonian fluid. Micromachines.

[CR25] Narayana Iyengar S, Kumar T, Mårtensson G, Russom A (2021). High resolution and rapid separation of bacteria from blood using elasto-inertial microfluidics. Electrophoresis.

[CR26] Nasser GA, El-Bab AMRF, Abdel-Mawgood AL, Mohamed H, Saleh AM (2019). CO_2_ laser fabrication of PMMA microfluidic double T-junction device with modified inlet-angle for cost-effective PCR application. Micromachines.

[CR27] Nasser GA, Fath El-Bab AMR, Mohamed H, Abouelsoud A. Low cost micro-droplet formation chip with a hand-operated suction syringe. Proceedings - 2018 IEEE 18th International Conference on Bioinformatics and Bioengineering, BIBE 2018, pp 73–78. 2019. 10.1109/BIBE.2018.00021

[CR28] Okello JL, El-Bab AM, Yoshino M, El-Hofy HA, Hassan MA (2022). Modelling of surface roughness in CO_2_ laser ablation of aluminium-coated polymethyl methacrylate (PMMA) using adaptive neuro-fuzzy inference system (ANFIS) in Volume 2B: Advanced Manufacturing. Am Soc Mech Eng.

[CR29] Olm F, Urbansky A, Dykes JH, Laurell T, Scheding S (2019). Label-free neuroblastoma cell separation from hematopoietic progenitor cell products using acoustophoresis - towards cell processing of complex biological samples. Sci Rep.

[CR30] Patil V, Tran K, Giselrød HR (2008). Towards sustainable production of biofuels from microalgae. Int J Mol Sci.

[CR31] Pødenphant M, Ashley N, Koprowska K, Mir KU, Zalkovskij M, Bilenberg B, Bodmer W, Kristensen A, Marie R (2015). Separation of cancer cells from white blood cells by pinched flow fractionation. Lab Chip.

[CR32] Robla J, Alguacil FJ, Dittami SM, Marie D, Deragon E, Guillebault D, Mengs G, Medlin LK, Robla J, Alguacil FJ, Dittami SM, Marie D (2021). Determination of the efficiency of filtration of cultures from microalgae and bacteria using hollow fiber filters. Environ Sci.

[CR33] Ruiz J, Olivieri G, De Vree J, Bosma R, Willems P, Reith JH, Eppink MHM, Kleinegris DMM, Wijffels RH, Barbosa MJ (2016). Towards industrial products from microalgae. Energy Environ Sci.

[CR34] Schaap A, Dumon J, Toonder JD (2016). Sorting algal cells by morphology in spiral microchannels using inertial microfluidics. Microfluid Nanofluid.

[CR35] Spilling K, Spilling K (2017). Basic Methods for Isolating and Culturing Microalgae. ) Biofuels from Algae Methods in Molecular Biology.

[CR36] Syed MS, Rafeie M, Vandamme D, Asadnia M, Henderson R, Taylor RA, Warkiani ME (2018). Selective separation of microalgae cells using inertial microfluidics. Biores Technol.

[CR37] Wan C, Alam MA, Zhao XQ, Zhang XY, Guo SL, Ho SH, Chang JS, Bai FW (2015). Current progress and future prospect of microalgal biomass harvest using various flocculation technologies. Biores Technol.

[CR38] Wang Y (2021). The automatic and high-throughput purification and enrichment of microalgae cells using deterministic lateral displacement arrays with different post shapes. J Chem Technol Biotechnol.

[CR39] Wang Y, Wang J, Xudong Jiang WW (2019). Dielectrophoretic separation of microalgae cells in ballast water in a microfluidic chip. Electrophoresis.

[CR40] Wang Y, Wang J, Cheng J, Zhang Y, Ding G, Wang X, Kang Y, Pan X (2020). Serial separation of microalgae in a microfluidic chip under inertial and dielectrophoretic forces. IEEE Sens J.

[CR41] Wang S, Liu Z, Wu S, Sun H, Wei J, Fan Z, Sui Z, Liu L, Pan X (2021). Microalgae separation by inertia-enhanced pinched flow fractionation. Electrophoresis.

[CR42] Yang Y, Pollard AM, Höfler C, Poschet G, Wirtz M, Hell R, Sourjik V (2015). Relation between chemotaxis and consumption of amino acids in bacteria. Mol Microbiol.

[CR43] Yu ZTF, Yong KMA, Fu J (2014). Microfluidic blood cell preparation: now and beyond. Small.

[CR44] Yuan D, Tan SH, Zhao Q, Yan S, Sluyter R, Nguyen NT, Zhang J, Li W (2017). Sheathless Dean-flow-coupled elasto-inertial particle focusing and separation in viscoelastic fluid. RSC Adv.

[CR45] Yuan D, Zhao Q, Yan S, Tang SY, Zhang Y, Yun G, Nguyen NT, Zhang J, Li M, Li W (2019). Sheathless separation of microalgae from bacteria using a simple straight channel based on viscoelastic microfluidics. Lab Chip.

[CR46] Zhang X, Zhu Z, Xiang N, Long F, Ni Z (2018). Automated microfluidic instrument for label-free and high-throughput cell separation. Anal Chem.

